# Bird dispersal of fruit-like yam bulbils: Is this a new form of Batesian mimicry?

**DOI:** 10.1073/pnas.2602471123

**Published:** 2026-03-19

**Authors:** Kenji Suetsugu, Steven D. Johnson

**Affiliations:** ^a^Department of Biology, Graduate School of Science, Kobe University, Kobe, Hyogo 657-8501, Japan; ^b^Institute for Advanced Research, Kobe University, Kobe, Hyogo 657-8501, Japan; ^c^Centre for Functional Biodiversity, School of Life Sciences, University of KwaZulu‐Natal Pietermaritzburg, Scottsville, Pietermaritzburg 3209, South Africa

Chen et al. provide an elegant illustration of how selection can circumvent a life-history constraint (1). *Dioscorea melanophyma* has largely lost sexual reproduction, yet birds are able to transport its clonal propagules over long distances. Jet-black, glossy bulbils provide berry-like cues that attract frugivores, leading to ingestion and dispersal. By combining avian color-space analyses with field observations, gut-passage viability tests, and movement-distance estimates, the authors link color signals to dispersal outcomes. This work shows how asexual lineages can regain frugivore-mediated dispersal without providing a conventional fruit reward (1).

At the same time, we note that the paper frames this interaction as “Batesian mimicry” and uses the term in the title, although the evidence does not clearly establish several defining features of Batesian mimicry. In its original sense, Batesian mimicry was characterized as a defensive phenomenon (protective mimicry) in which an undefended organism gains protection by resembling a defended model, thereby reducing predation risk (2). In recent decades, the term has also been applied to cases of inviting mimicry, such as rewardless flowers that imitate rewarding models to attract pollinators (3–7). Even under this broader usage, however, Batesian mimicry presumes model dependence and frequency dependence, typically mediated by conditioning or learned behavior in animals interacting with the model (4–7). The system described by Chen et al. instead aligns more closely with generalized food deception, a form of mimesis that deploys general signals exploiting the sensory biases of food-seeking animals without requiring a frequency-dependent association with one or more specific models (6, 8).

This interpretation is supported by two key results. First, bird visitation peaks from October to February, when fleshy fruits are scarce (1), making it unlikely that bulbil success depends on the local presence of a model. Second, cafeteria assays show that bulbil removal increases as berries become rarer (1), implying that success can be highest when real fruits are absent. This pattern is difficult to reconcile with model-dependent Batesian food-source mimicry, which is expected to rely primarily on learning and therefore to show a positive relationship between mimic fitness and model frequency (2, 4–6, 8).

Further analyses could help sharpen this distinction. Identifying candidate model species is an essential first step, although multiple models may be involved, analogous to multifarious Batesian mimicry (9). Tests of whether bulbil removal increases in proximity to, or in areas with higher abundances of, fruiting individuals of rewarding, dark-fruited species consumed by the same frugivores would provide more direct evidence for or against model dependence.

If future work demonstrates model dependence and clarifies the role of conditioning, the use of the term Batesian mimicry for this system could be justified. Regardless of whether the apparent mimesis of fruits by *Dioscorea* bulbils represents generalized food deception or Batesian mimicry, the findings of Chen et al. demonstrate that asexual lineages can attain frugivore-mediated dispersal without providing a typical fruit reward (1), thereby extending the concept of inviting mimicry in plants beyond flowers and fruits (4, 10).

## References

[1] Z. Chen, G. Chomicki, Y. Li, X. Peng, G. Chen, Berry Batesian mimicry enables bird dispersal of asexual bulbils in a yam. Proc. Natl. Acad. Sci. U.S.A. **123**, e2528094123 (2026). 41525478 10.1073/pnas.2528094123 PMC12818439

[2] G. D. Ruxton, T. N. Sherratt, M. Speed, Avoiding Attack: The Evolutionary Ecology of Crypsis, Warning Signals, and Mimicry (Oxford University Press, 2004).

[3] S. D. Johnson, Evidence for Batesian mimicry in a butterfly-pollinated orchid. Biol. J. Linn. Soc. **53**, 91–104 (1994).

[4] S. D. Johnson, F. P. Schiestl, Floral Mimicry (Oxford University Press, 2016).

[5] E. Newman, B. Anderson, S. D. Johnson, Flower colour adaptation in a mimetic orchid. Proc. R. Soc. B Biol. Sci. **279**, 2309–2313 (2012). 10.1098/rspb.2011.2375 PMC3350669 22298842

[6] C. I. Peter, S. D. Johnson, Mimics and magnets: The importance of color and ecological facilitation in floral deception. Ecology **89**, 1583–1595 (2008). 18589523 10.1890/07-1098.1

[7] F. P. Schiestl, S. D. Johnson, Pollinator-mediated evolution of floral signals. Trends Ecol. Evol. **28**, 307–315 (2013). 23480953 10.1016/j.tree.2013.01.019

[8] B. Anderson, S. D. Johnson, The effects of floral mimics and models on each others’ fitness. Proc. R. Soc. B Biol. Sci. **273**, 969–974 (2006). 10.1098/rspb.2005.3401 PMC1560233 16627282

[9] A. S. Papadopulos , Convergent evolution of floral signals underlies the success of Neotropical orchids. Proc. R. Soc. B Biol. Sci. **280**, 20130960 (2013). 10.1098/rspb.2013.0960 PMC3712443 23804617

[10] M.-F. Jin, X.-H. Cai, G. Chen, Seed dispersal by deception: A game between mimetic seeds and their bird dispersers. Plant Divers. **47**, 169–177 (2025). 40182487 10.1016/j.pld.2024.07.006 PMC11962984

